# Near infrared spectroscopy‐derived muscle oxidative capacity correlates to oxidative metabolic predictors of performance in highly trained female middle‐distance runners

**DOI:** 10.14814/phy2.71047

**Published:** 2026-08-09

**Authors:** Mira I. Schoeberlein, Louis D. Hunter, Emma W. Richardson, Brian A. Irving, Brad W. Wilkins

**Affiliations:** ^1^ Oregon Performance Research Laboratory, Department of Human Physiology University of Oregon Eugene Oregon USA; ^2^ School of Kinesiology Louisiana State University Baton Rouge Louisiana USA; ^3^ Department of Kinesiology University of Virginia Charlottesville Virginia USA

**Keywords:** female athletes, muscle oxidative capacity, near infrared spectroscopy, physiological predictors of performance

## Abstract

Investigate the relationship between muscle oxidative capacity, indices of O_2_ metabolism (maximal capacity and sustainable steady state), and severe domain performance in highly trained female middle‐distance runners. Incremental treadmill test determined maximal oxygen uptake (V̇O_2max_) and lactate turn point (LTP). Critical speed (CS) was calculated from historical race and training data. Time‐to‐exhaustion (TTE) trials were run at a speed predicted to elicit exhaustion in 4 min. Muscle oxidative capacity (*rectus femoris*) was measured via muscle oxygen uptake (mV̇O_2_) from near infrared spectroscopy (NIRS) deoxygenation slopes during intermittent arterial cuff occlusions (8 × 5 s ON/5 s OFF) following light exercise. Slopes were fit to a mono‐exponential model to calculate recovery rate (*k*). Muscle oxidative capacity was highly correlated to V̇O_2max_ (*r* = 0.71, *p* = 0.002), LTP (*r* = 0.67, *p* = 0.005), and estimated CS (*r* = 0.66, *p* = 0.005). However, muscle oxidative capacity was not correlated to TTE performance (*r* = 0.13, *p* = 0.622). NIRS‐derived muscle oxidative capacity is strongly associated with maximal and sustainable oxidative metabolic rates in highly trained female runners but appears independent of the finite energy capacity required during a severe‐domain performance.

## 1 INTRODUCTION

Maximal rates of mitochondrial oxidative metabolic flux in skeletal muscle are highly adaptable and strongly associated with maximal whole‐body aerobic power and exercise performance (Gollnick et al., 1973; Holloszy, 1967; Hoppeler et al., 1985). Traditionally, maximal rates of skeletal muscle oxidative metabolic flux have been assessed using invasive muscle biopsy samples combined with ex vivo techniques, such as enzyme activity assays (Gollnick et al., 1973; Holloszy, 1967), permeabilized muscle fibers (Garcia‐Roves et al., 2025), or isolated mitochondrial preparations (Chance & Williams, 1955). Alternatively, in vivo measurements of maximal muscle oxidative energy flux from Phosphocreatine (PC_r_) recovery kinetics have been obtained using more costly approaches, such as ^31^P‐magnetic resonance spectroscopy (^31^P‐MRS) (Larson‐Meyer et al., 2001; McCully et al., 1993; Paganini et al., 1997). Over the past two decades, a non‐invasive and less costly approach has emerged utilizing near‐infrared spectroscopy (NIRS) to measure the rate of recovery of muscle oxygen consumption (mV̇O_2_) following a brief bout of exercise (Hamaoka et al., 2011; Ryan, Southern, et al., 2013; Zuccarelli et al., 2020).

The NIRS‐derived recovery rate constant (*k*) is a valid measure of maximal muscle oxidative kinetics compared to traditional cellular techniques (Ryan, Southern, et al., 2013) and in vivo measurements with ^31^P‐MRS (Hamaoka et al., 2011), and it demonstrates good repeatability (Hanna et al., 2021). The NIRS‐derived method describes the maximal rate of muscle V̇O_2_ off‐kinetics but is almost exclusively referred to in the literature as muscle oxidative capacity, despite being related to the maximal rate of muscle oxygen consumption and not a capacity. To be consistent with common nomenclature throughout the literature, we will refer to this measure as muscle oxidative capacity. The NIRS approach has been widely applied to examine skeletal muscle oxidative capacity across a range of populations, including healthy individuals (Ryan et al., 2012; Ryan, Brizendine, & McCully, 2013), trained athletes (Batterson et al., 2020; Brizendine et al., 2013; Possamai et al., 2023), and patients with chronic obstructive pulmonary disease (Adami et al., 2017), among other conditions (Adami & Rossiter, 2018; Davis et al., 2024). A non‐invasive NIRS‐derived measurement of skeletal muscle oxidative capacity is also correlated with whole‐body maximal oxygen uptake (V̇O_2max_) as determined by pulmonary gas exchange (Beever et al., 2020; Guzman et al., 2020; Lagerwaard et al., 2019; Possamai et al., 2023). However, this relationship between V̇O_2max_ and muscle oxidative capacity appears to be dependent on the muscle interrogated (Beever et al., 2020; Lagerwaard et al., 2019) and has not been demonstrated unequivocally (de Aguiar et al., 2022; Venckunas et al., 2024).

Despite the importance of skeletal muscle oxidative capacity in our understanding of maximal whole‐body aerobic power (e.g., V̇O_2max_), its relationship to the highest sustainable metabolic rate remains poorly understood. The highest steady‐state metabolic rate represents the maximal exercise intensity at which oxygen supply remains matched to oxygen utilization (Kirby, Clark, et al., 2021; Matthews et al., 2023) and is therefore highly dependent on mitochondrial oxidative flux (Jones et al., 2008). This maximal metabolic steady state (MMSS) delineates the boundary between the heavy and severe exercise intensity domains, where exercise performed above this boundary leads to progressive metabolic instability and eventual exhaustion (Jones et al., 2019; Poole et al., 1988; Wasserman et al., 1973). To our knowledge only one study has explored the relationship between an index of MMSS, specifically maximal lactate steady state, and muscle oxidative capacity of the *vastus lateralis* in a group of trained male rowers (Possamai et al., 2023). In fact, data describing muscle oxidative capacity in highly trained athletes remain relatively sparse (Batterson et al., 2020; Brizendine et al., 2013; Possamai et al., 2023) and studies examining relationships between muscle oxidative capacity and oxidative predictors of performance in trained female runners do not exist. Middle‐distance runners, and female runners in particular, remain underrepresented in the literature. With the recent high‐profile interest in the potential for a female runner to break the sub‐4‐min mile barrier (Osborne et al., 2025), it is necessary to describe the physiological characteristics a female runner must possess to accomplish this goal.

The primary objective of this investigation was to examine the relationship between skeletal muscle oxidative capacity in the *rectus femoris* (a biarticular muscle highly active during running) and two independent oxidative metabolic predictors of performance, namely V̇O_2max_ and MMSS, in highly trained female middle‐distance runners. An additional aim was to correlate muscle oxidative capacity in the *rectus femoris* to running performance at speeds designed to elicit fatigue in 4‐min. We hypothesized that NIRS‐derived recovery rate constants, reflecting skeletal muscle oxidative capacity, would be positively correlated with both the maximal rate of oxidative metabolism (V̇O_2max_) and the maximal steady state rate of oxidative metabolism (or MMSS). We further hypothesized that muscle oxidative capacity would be positively correlated with time to exhaustion (TTE) during severe domain running at speeds intended to elicit failure in 4‐min.

## 2 METHODS

### 2.1 Participants and ethical approval

Experimental procedures and protocols received institutional review board approval from the University of Oregon's Institutional Review Board (protocol STUDY1566) and conformed to the standards set by the Declaration of Helsinki. Participants were informed of all procedures and any potential risks of participating in the study and provided written informed consent prior to study enrollment. Study participants were recruited from the University of Oregon student population and the greater Eugene community through recruitment flyers and word of mouth. All participants were non‐obese (BMI <30 kg/m^2^), normotensive (resting blood pressure <120/80 mmHg per American Heart Association guidelines), and were not taking any medications (apart from oral contraceptives).

Sixteen highly trained female runners (identified via self‐reported sex assigned at birth) aged 18–30 years, and capable of running a six‐minute mile or faster, volunteered to participate in this study. The six‐minute mile inclusion criteria ensured recruitment of a highly trained, middle‐distance focused population. All participants met Tier 2 – Tier 4 training and performance classification as defined by McKay et al., with the majority of participants fitting the Tier 3 criteria (McKay et al., 2021). Of the sixteen female runners, five had natural menstrual cycles, ten used intrauterine devices, and one used a combination birth control pill. We did not control for menstrual cycle phase.

All participants were instructed to abstain from caffeine and food for at least 2‐h, and strenuous exercise, alcohol, recreational drugs, nutritional supplements, vitamins, over the counter medications, and prescription drugs (except hormonal contraceptives) for 24‐h before each visit. Outside of these abstentions, they were instructed to maintain consistent activity levels, diet, and caffeine intake throughout the study. Participants were also asked to wear the same running shoes for all trials.

### 2.2 Experimental approach

Participants visited the laboratory on two separate occasions. On visit 1, they provided informed written and verbal consent to participate in the study prior to a graded exercise treadmill test to measure maximal oxygen uptake (V̇O_2max_) and lactate turn point (LTP). Before visit 1, participants provided self‐reported records for race distances between 800 meters and 10,000 meters (if they existed), or training data from 800 m to 10,000 m within the past year, which were used to estimate each participant's speed–duration relationship and critical speed (CS). Visit 2 included a non‐invasive measurement of muscle oxidative capacity followed by a time to exhaustion (TTE) running trial at a speed based on their estimated CS and LTP.

### 2.3 Measurements

Experimental sessions were conducted in a temperature‐controlled laboratory (~20°C). All runs were completed on a treadmill at 1% incline (Pro XL, Woodway USA Inc., Waukesha, WI).

Heart rate was continuously measured via telemetry (Model H10, Polar Electro, Kemple, Finland). Pulmonary gas exchange for oxygen uptake (V̇O_2_) and carbon dioxide production (V̇CO_2_) was determined (K5, COSMED, Rome, Italy) from minute ventilation (V̇_E_) and expired gas concentrations (FEO_2_ and FECO_2_) sampled from a mixing chamber. Complete system calibration procedures for expired air flow rate (3 L syringe) and respiratory gas concentrations (known O_2_ and CO_2_ concentrations) were performed per system instructions before each experiment. Mixed venous blood samples were obtained via finger prick and analyzed for blood lactate concentration in duplicate using a portable lactate analyzer (Lactate Plus, Nova Biomedical, Waltham, MA, USA).

Critical speed and D′ (finite energy reserve; measured in m) were calculated using historical race or personal records (obtained within 1 year) over a range of distances (800 m–10,000 m) using a two‐parameter speed duration relationship model (D = CS × t + D′), where D represents distance and *t* represents time (Jones et al., 2019). At least three data points were used for each participant, often more, over this range of distances. We have previously demonstrated previous race records over these distances accurately represent CS and D′ and can be highly predictive of performance outcomes in the context of a race (Kirby, Winn, et al., 2021).

Height was measured via a stadiometer (Seca 213, Hamburg, Germany). Body mass via an electronic scale (PA‐550XL, PatientAid). Skinfold thickness was measured at the location where the NIRS sensors were placed (right leg *rectus femoris*) by calipers (Lange Calipers, Perform Better, West Warwick, RI) and averaged across 3 measurements. Adipose tissue thickness (ATT) was calculated as one half of the skinfold thickness.

### 2.4 Ramp treadmill protocol

Maximal oxygen uptake and LTP were determined via graded treadmill test to volitional exhaustion. Briefly, a short, self‐selected warm‐up on the treadmill was followed by the graded running test protocol at an individualized starting speed based on each participant's easy running pace and estimated CS. Each stage was 3 min in duration, where the belt speed increased by 1 kph for the first three stages, then 0.5 kph at each subsequent stage until participants reached volitional exhaustion. With 30 s left in each stage, participants straddled the treadmill belt, and blood lactate samples were obtained. Expired respiratory gases and HR were measured continuously.

### 2.5 Muscle oxidative capacity protocol

Muscle oxidative capacity was determined using intermittent arterial occlusions following a brief bout of exercise, using a modified Mito6 protocol (Hanna et al., 2021; Sumner et al., 2020), with NIRS sensors (PortaLite, Artinis Medical Systems, Netherlands) to measure oxy‐, deoxy‐ and total hemoglobin/myoglobin content and tissue saturation index (%TSI) in *rectus femoris* (Ryan et al., 2012; Ryan et al., 2014; Zuccarelli et al., 2020). Importantly, the *rectus femoris* is a biarticular muscle, crossing both the hip and the knee, and is highly active during running. The NIRS sensor was positioned longitudinally and placed halfway between the inguinal line and the patella, centrally over the *rectus femoris* of the right leg, and secured with adhesive tape. The NIRS sensor (PortaLite) is a portable, continuous wave, spatially resolved device that quantifies tissue oxygenation via a modified Beer–Lambert algorithm, emitting NIR light at 760 and 850 nm across three channels (30, 35, and 40 mm separation distances between source and detectors) with a differential pathlength factor (DPF) of 4. Relative concentrations of oxy‐(hemoglobin + myoglobin) and deoxy‐(hemoglobin + myoglobin) were recorded ~1.5–2 cm beneath the probe relative to a resting baseline set to zero. Total hemoglobin and myoglobin (deoxy + oxy) and the Hb difference (oxy – deoxy) were calculated. The %TSI was measured using spatially resolved spectroscopy (Ferrari et al., 2004).

While seated in a phlebotomy chair with legs supported in an extended position and relaxed, participants were also fitted with a rapid‐inflation thigh cuff (SC12D; Hokanson, Bellevue, WA, USA) connected to an electronic inflator (E20; Hokanson) set to inflate to a pressure of ~250 mmHg to achieve total arterial occlusion (AO).

**FIGURE 1:**
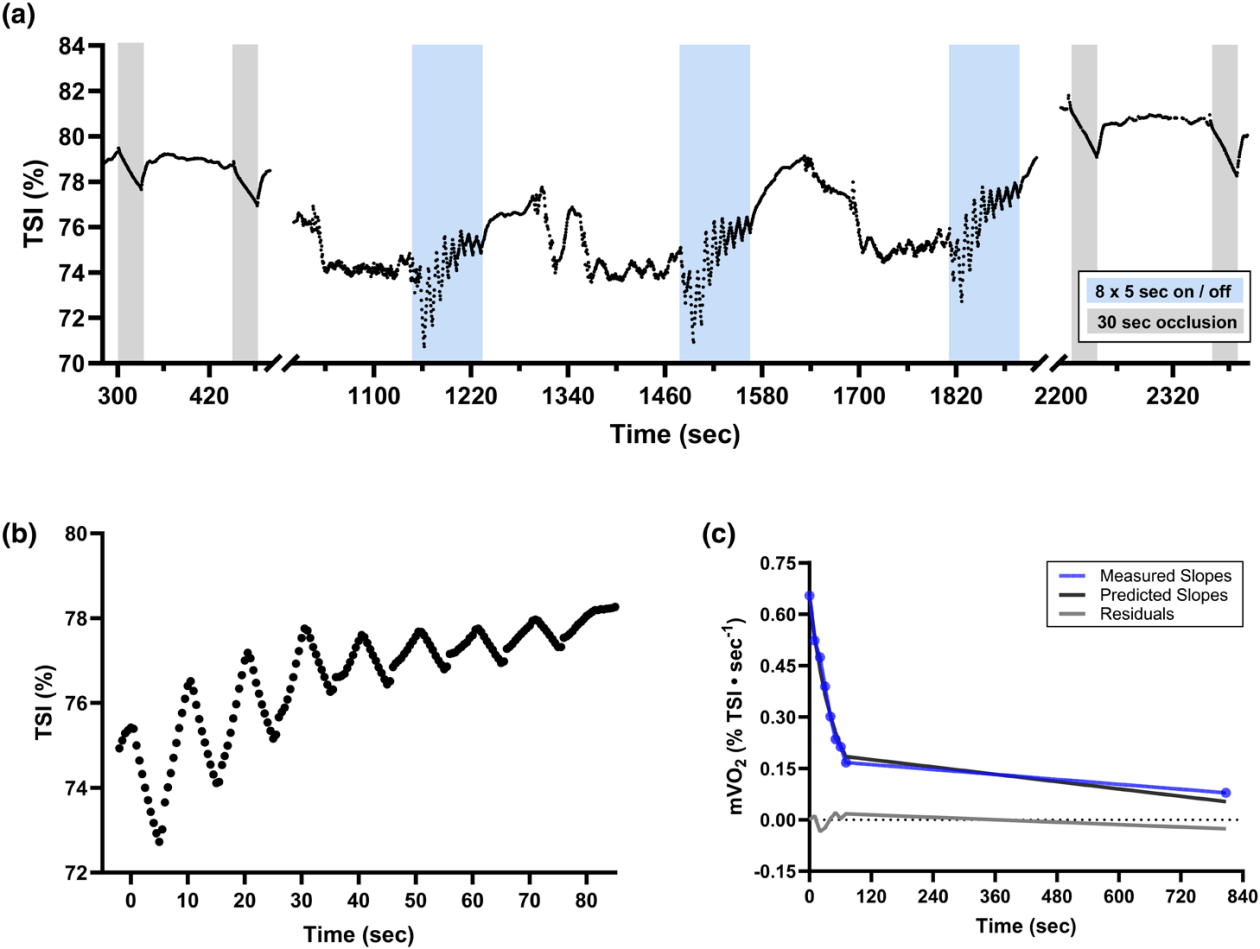
Representative tracing from a single participant of the muscle oxidative capacity protocol. (a) Tissue saturation index (%TSI) throughout the entire protocol, consisting of four 30‐s occlusions and three exercise‐intermittent occlusion bouts. (b) %TSI response during the intermittent occlusions; mV̇O_2_ was calculated as the negative rate of change of %TSI signal during each occlusion. (c) The average mV̇O_2_ measurements from the intermittent occlusions were used to model the mono‐exponential post‐exercise mV̇O_2_ recovery.

The protocol consisted of a series of AO's to determine the rate of muscle oxygen consumption (mV̇O_2_), defined as the negative linear slope of the NIRS‐derived TSI signal during the AOs, which represents mV̇O_2_ within the underlying muscle tissue. The protocol began with two baseline measurements of mV̇O_2_ for 30 s with 2 min of rest between each AO. Following baseline measurements, participants moved to a cycle ergometer (Lode Excalibur Sport, Groningen, the Netherlands) and performed 2 min of low‐intensity cycling at 1.5 W/kg and ~75 rpm to increase muscle blood flow and mV̇O_2_. Cycling exercise allowed standardization of work between participants. Participants were then quickly transferred from the bike to the identical seated position, and recovery kinetics of mV̇O_2_ were measured with a series of 8 AO's, with reperfusion timed at 5 s on/5 s off. The exercise and eight 8 AO's were repeated two more times with 2 min of rest between the end of the occlusions and the next exercise bout. After the final exercise‐intermittent occlusion cycle, a 5‐min rest was followed by 2 more measurements of baseline mV̇O_2_. Figure 1 depicts a representative tracing of the NIRS‐derived tissue saturation index (%TSI) signal during the muscle oxidative protocol.

NIRS data were collected at 50 Hz and down‐sampled to 2 Hz for analysis. As noted above, mV̇O_2_ was calculated as the negative slope of the %TSI signal during the AO via linear regression. The post‐exercise mV̇O_2_ recovery was modeled with a mono‐exponential function according to the formula below (Ryan, Brizendine, & McCully, 2013).

$$y\left(t\right)=\mathrm{End}-\Delta \times e^{-\mathrm{kt}}$$

where $y\left(t\right)$ is the mV̇O_2_ at time *t*, End is the mV̇O_2_ immediately after exercise, ∆ is the change in mV̇O_2_ from immediately post exercise to full recovery, k is the recovery rate constant, and the recovery time constant, τ=1/k. The resulting rate constant (*k*) and time constant (τ) were used as indices of muscle oxidative capacity. For the modeling, the mV̇O_2_ slopes were averaged across the repeated intermittent occlusion trials. To avoid erroneous slopes across the three trials, the coefficients of variation (CV) were assessed and optimized to be below 20% (mean CV = 11.82% ± 2.73%). The optimization identified erroneous slopes as those which resulted in a CV across the three trials greater than 30%, and the removal of the slope resulted in a measurable decrease in the CV (Southern et al., 2014). Across the 448 measured slopes, 34 were identified as erroneous (< 10% of measured slopes).

### 2.6 TTE protocol

On visit 2, participants completed a treadmill TTE running trial at a fixed treadmill speed. Treadmill speed was prescribed based on their individual speed duration parameters (CS and D′) using the equation: *T*_lim_ = D′/(S − CS), where *T*_lim_ is time to exhaustion and S was the prescribed treadmill speed (Fukuba & Whipp, 1999). The treadmill speed (S) was set based on the calculation that *T*_lim_ be approximately 4 min. Participants were instructed to complete their typical pre‐race warm‐up and stretching routine. Participants were fitted with a safety harness (Woodway USA Inc., Waukesha, WI) secured around the chest, which is tethered to the treadmill via a “shut‐off switch” that automatically stops the treadmill belt. During the trial, participants were instructed and verbally motivated to continue running at the prescribed speed for as long as possible, while blinded to both the set speed and the elapsed time. Exhaustion typically occurred between 3.5 and 4.5 min. Oxygen uptake, expired gases, and HR were measured continuously. Blood lactate samples were collected immediately before and after the exercise bout.

### 2.7 Data analysis

Oxygen uptake and V̇CO_2_ were collected at 10‐s intervals. To ensure no single erroneous value (either high or low) significantly biased the V̇O_2max_, maximal V̇O_2_ was determined as the highest average V̇O_2_ over 30 s at the end of the ramp treadmill protocol or during the TTE trial. Blood lactate concentration was plotted against treadmill speed to identify running speed associated with LTP. The first lactate threshold was defined as the initial increase in blood lactate concentration above the baseline value of approximately 2 mM, and LTP was determined by the subsequent sudden and sustained increase in blood lactate concentration (Smith & Jones, 2001). Time trial performance was calculated as the total distance traveled until exhaustion during the TTE.

### 2.8 Statistics

Data are reported as group mean ± SD. Normality of the data was assessed by the Shapiro Wilk test, with no data requiring transformation. Relationships between indices of muscle oxidative capacity (τ and *k*) and V̇O_2max_, LTP, estimated CS, and TTE Distance were assessed using Pearson correlation and linear regression. Both *r* values and *R* ^2^ values are presented where appropriate. Statistical significance was set a priori at *p* < 0.05. All statistical analysis was completed using GraphPad Prism (GraphPad Software, San Diego, California, USA).

## 3 RESULTS

Participant characteristics are presented in Table 1 (mean ± SD).

**TABLE 1:** Participant characteristics (mean ± SD).

	Group
*n* =	16
Age (year)	27 ± 5
Height (cm)	163.9 ± 5.0
Weight (kg)	59.1 ± 7.6
Skinfold thickness (mm)	21.6 ± 6.9
ATT (mm)	10.8 ± 3.4

Abbreviation: ATT, adipose tissue thickness.

### 3.1 Muscle oxidative capacity and exercise metrics

The average mV̇O_2_ recovery time constant (*τ*) was 36.6 ± 7.2 s and the average mV̇O_2_ recovery rate constant (*k*) was 1.702 ± 0.360 min^−1^. The average V̇O_2max_ was 59.7 ± 6.2 mL·kg^−1^·min^−1^. The speeds associated with MMSS as determined by LTP and estimated CS were 15.0 ± 1.5 kph and 14.8 ± 1.6 kph, respectively. The mean estimated D′ was 0.204 ± 0.051 km. Lactate turn point was highly correlated to both V̇O_2max_ (*r* = 0.84; *p* < 0.001) and estimated CS (*r* = 0.91; *p* < 0.001). Estimated CS was also highly correlated V̇O_2max_ (*r* = 0.84; *p* < 0.001).

**FIGURE 2:**
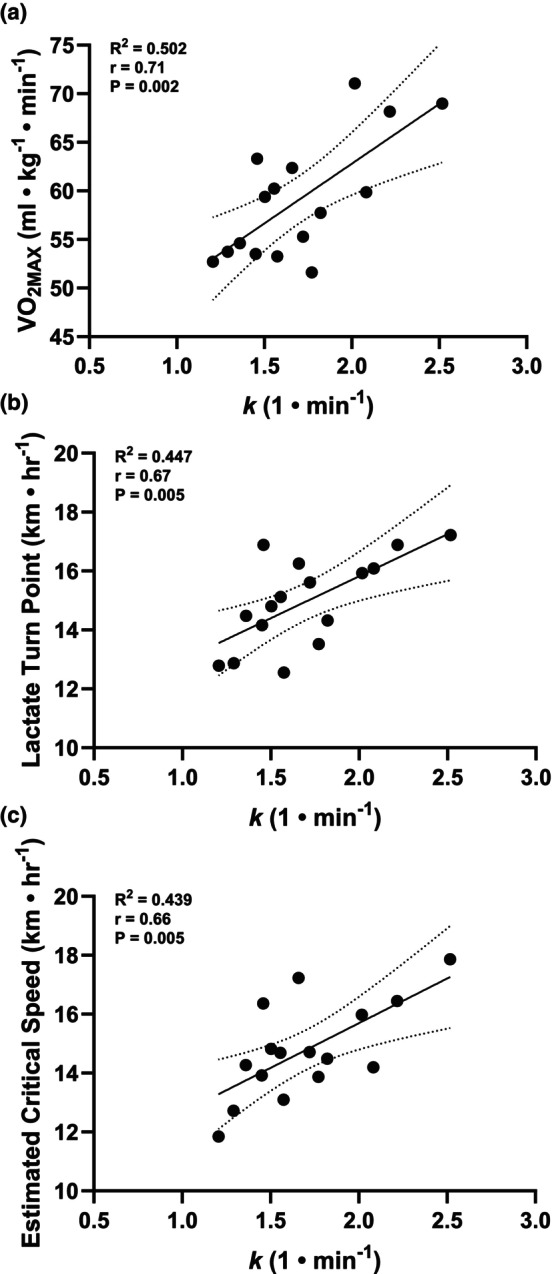
Graphical representation (with regression lines) for (a) V̇O_2max_, (b) lactate turn point, and (c) estimated critical speed relationship to NIRS‐derived muscle oxidative capacity recovery rate constant (*k*) (*n* = 16).

A graphical representation of the relationship between oxidative capacity (*k*) and oxidative exercise metrics is represented in Figure 2a–c. The results of the Pearsons correlation between the NIRS indices of muscle oxidative capacity (both *k* and *τ*) and all exercise metrics are summarized in Table 2. Maximal oxygen uptake (V̇O_2max_) and both measures of MMSS, LTP and estimated CS, are all strongly correlated to muscle oxidative capacity (expressed as *k* and *τ*; Table 2 and Figure 2).

**TABLE 2:** Pearsons correlation between NIRS indices of muscle oxidative capacity and the exercise metrics.

	*k*		*τ*	
	*r*	*p*	*r*	*p*
V̇O_2max_	0.71	**0.002**	−0.68	**0.004**
LTP	0.67	**0.005**	−0.67	**0.005**
Estimated CS	0.66	**0.005**	−0.67	**0.004**
TTE Distance	0.13	0.622	−0.21	0.443

*Note*: The bolded format is used to denote statistically significant *p*‐values from non‐significant values.

Abbreviations: CS, critical speed; LTP, lactate turn point; V̇O_2max_, maximal oxygen uptake.

### 3.2 Relationship between muscle oxidative capacity and TTE performance

**FIGURE 3:**
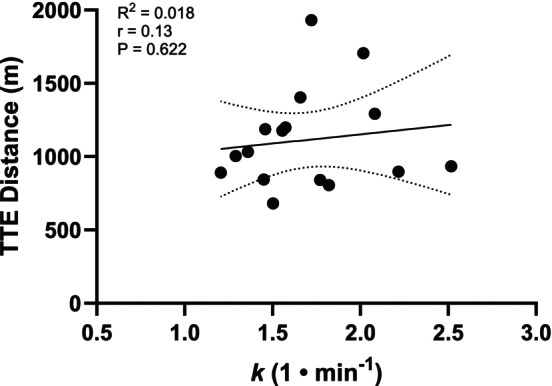
Graphical representation (with regression lines) for time to exhaustion (TTE) performance (distance run) relationship to NIRS‐derived muscle oxidative capacity recovery rate constant (*k*) (*n* = 16).

Average TTE was 238.9 ± 75.1 s, demonstrating success in prescribing individual speeds to elicit exhaustion in ~4 – min. Group TTE performance, as measured by running distance to exhaustion at a set speed, was 1114.0 ± 339.4 m. During TTE, V̇O_2_ reached 103% ± 7% of V̇O_2max_ measured during the incremental test, HR reached 184 ± 7 bpm, and post TTE venous blood lactate reached 8.6 ± 2.1 mM. The relationship between muscle oxidative capacity and TTE performance is presented in Figure 3. There was no correlation between TTE performance and muscle oxidative capacity (Figure 3 and Table 2). By design, CS and D′ combined determined speed for the TTE, however the magnitude of estimated D′ demonstrated no correlation (*r* = 0.36; *p* = 0.18) with TTE performance.

## 4 DISCUSSION

The primary goal of this investigation was to explore the relationship between muscle oxidative capacity and key oxidative metabolic predictors of endurance performance in highly trained female middle‐distance runners. In line with our hypothesis, the NIRS‐derived mV̇O_2_ recovery rate constant (*k*), an index of muscle oxidative capacity, was strongly correlated with whole‐body maximal oxidative metabolic rate (V̇O_2max_; Figure 2a) and with measures of the highest steady‐state oxidative metabolic rate (MMSS), assessed by both LTP and an estimated CS (Figure 2b,c). In contrast to our second hypothesis, muscle oxidative capacity was not correlated with middle‐distance, severe domain running performance (Figure 3).

### 4.1 Muscle oxidative capacity and oxidative predictors of performance

Several studies have demonstrated strong associations between NIRS‐derived muscle oxidative capacity and V̇O_2max_ (Beever et al., 2020; Guzman et al., 2020; Lagerwaard et al., 2019; Possamai et al., 2023), although this relationship appears to be muscle specific (Beever et al., 2020; Lagerwaard et al., 2019). Maximal oxygen uptake depends on both oxygen transport to the working muscle and the capacity of skeletal muscle to utilize that oxygen for ATP production through mitochondrial oxidative phosphorylation (Levine, 2008). Although convective oxygen transport may ultimately limit V̇O_2max_ (Levine, 2008), high skeletal‐muscle mitochondrial capacity is essential for sustaining high rates of oxidative energy flux (Gollnick et al., 1973; Holloszy, 1967). It is therefore not surprising that the cohort of highly trained female middle‐distance runners in the present study exhibited relatively high V̇O_2max_ values (59.7 ± 6.2 mL·kg^−1^·min^−1^) and that muscle oxidative capacity was strongly correlated with V̇O_2max_ (Figure 2a). This observation is consistent with previous work demonstrating strong relationships between NIRS‐derived measures of muscle oxidative capacity in *vastus lateralis* and V̇O_2max_ in trained athletes (Beever et al., 2020; Possamai et al., 2023). In contrast, Beever et al. (Beever et al., 2020) observed muscle oxidative capacity in the *medial gastrocnemius* did not correlate with V̇O_2max_ obtained during cycling exercise. This suggests the importance of sports specificity for measurements of muscle oxidative capacity and its relationship to maximal oxidative metabolic parameters.

Along with running economy, running performance is largely determined by V̇O_2max_ and the fraction of V̇O_2max_ that can be sustained (or the speed at MMSS) over the course of a race (Jones et al., 2021; Joyner, 1991). Middle‐distance race performance requires very high rates of oxygen uptake, as the oxygen demand of these events typically exceeds an athlete's V̇O_2max_. Consequently, a higher V̇O_2max_ and enhanced muscle oxidative capacity can reduce the oxygen deficit incurred during high‐intensity exercise (Billat et al., 2009). In addition, a higher speed associated with MMSS should enable faster running speeds at a given rate depletion of the finite energy capacity above the MMSS (Osborne et al., 2025).

There are several metrics commonly used to identify the external work rate associated with MMSS, including critical power (CP) (Jones et al., 2019), CS during running (Smith & Jones, 2001), and maximal lactate steady state (Iannetta et al., 2018). Lactate turn point has also been shown to highly correlate with traditional measures of CS and represents a reasonable physiological surrogate for MMSS when more time intensive protocols are not practical (Sablain et al., 2025; Smith & Jones, 2001; Valenzuela et al., 2021). In addition to LTP, we estimated CS using previous race records across various distances (800 m – 10 km), which can be a highly effective method predicting a subsequent race performance (Kirby, Winn, et al., 2021), especially a time trial performance. Consistent with previous findings, speed at LTP (15.0 ± 1.5 kph) and speed at estimated CS (14.8 ± 1.6 kph) were highly correlated (r = 0.91, *p* < 0.001), increasing our confidence that, combined, they accurately represent individual participants' MMSS.

To our knowledge, only one study has examined the relationship between muscle oxidative capacity and physiological indices that identify MMSS in a cohort of male rowers (Possamai et al., 2023). Muscle oxidative capacity was found to be highly related to both V̇O_2max_ and the power associated with MLSS in the cohort of male rowers (Possamai et al., 2023). The present study confirmed that, in a cohort of highly trained female middle‐distance runners, surrogate measures of MMSS are highly correlated to muscle oxidative capacity (Figure 2b,c). Although we did not necessarily expect there to be a difference based on sex between the present study and that of Possamai et al. (2023), it is critical to increase the representation of female athletes in the literature. The relationship between muscle oxidative capacity and MMSS is physiologically plausible, as mitochondrial function and ATP production via oxidative phosphorylation are fundamental determinants of MMSS (Conley et al., 2001; Jones et al., 2008). The relationship between V̇O_2max_ and muscle oxidative capacity also appears to be muscle specific (Beever et al., 2020; Lagerwaard et al., 2019) and has not been consistently demonstrated (de Aguiar et al., 2022; Venckunas et al., 2024). The findings here demonstrate this muscle specificity, where muscle oxidative capacity in the *rectus femoris*, a biarticular muscle highly active during running, is highly correlated with both V̇O_2max_ and MMSS obtained during running activities.

Taken together, this study is the first to demonstrate strong relationships between muscle oxidative capacity in a highly running specific muscle, like the *rectus femoris*, and both maximal oxidative metabolism (V̇O_2max_) along with two surrogate measures of the highest steady state oxidative metabolism (MMSS; Figure 2a–c) in a cohort of highly trained female middle‐distance runners.

### 4.2 Severe domain performance

With the recent interest in the potential for a female to run a mile in under 4‐min, there is a need to better understand the physiological determinants of middle‐distance running performance. In particular, to better understand the potential for a female runner to possess the physiological requirements to run a mile in under 4‐min (Osborne et al., 2025). As discussed previously, middle‐distance race performance requires rates of oxygen uptake that typically exceed the runner's V̇O_2max_. A higher V̇O_2max_ and a greater muscle oxidative capacity can reduce the oxygen deficit incurred during high‐intensity exercise (Billat et al., 2009). Therefore, our goal was to explore the relationship between muscle oxidative capacity and middle‐distance race performance in female runners. In contrast to our hypothesis, muscle oxidative capacity did not correlate with TTE running performance in a severe domain trial designed to elicit exhaustion in about 4‐min. It is interesting to note that Possamai et al. (2023) also found that 2000 m rowing performance, which is severe domain exercise, in their male cohort was not associated with muscle oxidative capacity. Combined with the results from the present study of female middle‐distance runners, it suggests that non‐oxidative metabolic factors, or substrate level phosphorylation, may be indicative of exercise performance completed entirely in the severe domain.

The treadmill speed for the TTE in the present study was prescribed based on each participant's speed–duration parameters, as determined from previous race performances and recent training times within the last year. The estimated CS represents the asymptote and D′ represents the curvature constant of the speed‐time relationship determined for each participant. Specifically, TTE running speed was selected such that the predicted time to exhaustion (*T*_lim_) would occur at approximately 4‐min. Time trial performance at this intensity requires running well above V̇O_2max_ and therefore demands energy production above MMSS and complete utilization of the finite energy reserve (D′), which reflects substrate‐level phosphorylation (Billat et al., 2009). Although a high CS is advantageous for middle‐distance performance, both the magnitude of D′ and the athlete's ability to fully utilize this energy reserve will significantly influence performance during severe‐domain exercise. Muscle acidification can also directly impair mitochondrial oxidative capacity (Jubrias et al., 2003) which could cause a dissociation between muscle oxidative capacity and performance in the severe domain. Accordingly, the absence of a relationship between muscle oxidative capacity and time‐to‐exhaustion performance in the severe domain (Figure 3) is not entirely surprising. The present data suggest that high muscle oxidative capacity primarily reflects the ability to sustain high steady‐state rates of mitochondrial energy transfer (Figure 2a,b) but appear independent of TTE performance (Figure 3).

### 4.3 Considerations and limitations

By design, running speed for the TTE trial was individually set to elicit exhaustion in ~4‐min. Although variable, average TTE was 238.9 ± 75.1 s demonstrating success in prescribing the running seed to elicit exhaustion in ~4‐min. Participants were blinded to elapsed time and distance throughout the constant speed TTE trial, creating an ‘open‐loop’ TTE trial where no feedback was provided (St Clair Gibson et al., 2001). Physiological strain may be higher during constant work‐rate performance trials (Lander et al., n.d.) and the impact of a physiological intervention (e.g. hypoxia) is higher during constant work‐rate TTE trials (Amann et al., 2008). Further, ‘open loop’ constant work‐rate trials in the absence of any feedback, which was the case here, can have a significant influence on TTE performance (Couto et al., 2022; Smits et al., 2016). This may have contributed to the lack of a correlation between the magnitude of D′ and TTE performance (*r* = 0.36; *p* = 0.18). The average distance covered during the TTE trial in the present study was 1114.0 ± 339.4 meters. It is unknown if muscle oxidative capacity would be related to performance in a ‘closed‐loop’ time‐trial of a set distance, 1500 m or 1 mile (1609 m) for example.

Critical speed was not directly determined using traditional laboratory‐based protocols in the present study. Instead, estimated CS values were derived from previous race performances and recent training data within the last year using individual speed‐time relationships. Previous work has demonstrated that race records can provide highly accurate predictions of endurance performance outcomes (Kirby, Winn, et al., 2021). Furthermore, estimated CS (14.8 ± 1.6 kph) was very similar to the physiological surrogate LTP (15.0 ± 1.5 kph) and the two measures were strongly correlated (*r* = 0.92, *p* < 0.001), suggesting our estimates of CS were accurate.

Although LTP may vary across individuals, it represents a practical physiological surrogate for MMSS (Smith & Jones, 2001; Valenzuela et al., 2021). Given that NIRS‐derived muscle oxidative capacity reflects mitochondrial oxidative function, it should theoretically be strongly associated with determinants of the highest steady‐state oxidative metabolic rate. Despite the methodological limitations associated with estimating CS, the strong relationships observed in the present study clearly support this physiological link.

Menstrual cycle phase was not controlled for when scheduling the measurement of muscle oxidative capacity. Menstrual cycle phase has recently been shown to impact absolute muscle oxygenation values (Baker et al., 2026); however, this report was not specific to muscle oxidative capacity measures and as the determination of muscle oxidative capacity relies on calculated slopes, changes in absolute values will likely have limited influence on the overall mV̇O_2_ recovery kinetics. Females tend to have greater adipose tissue thickness (ATT) than men (Craig et al., 2017), which may result in poor or blunted NIRS‐derived muscle oxygenation measurements (van Beekvelt et al., 2001). However, the ATT of our participants (Table 1) is well within the acceptable range given the source‐detector distance of the NIRS device used (McManus et al., 2018; Ryan et al., 2012).

## 5 CONCLUSION

Muscle oxidative capacity was strongly associated with both maximal (V̇O_2max_) and highest sustainable oxidative metabolic rates (MMSS) in highly trained female middle‐distance runners. This suggests that NIRS‐derived measures of muscle oxidative capacity reflect a key physiological determinant of mitochondrial energy flux during endurance exercise. However, muscle oxidative capacity did not predict performance during a blinded ‘open loop’ TTE trial in the severe domain, potentially due to the additional influence of finite energy reserves. These findings demonstrate skeletal muscle oxidative capacity, in the *rectus femoris*, is an important determinant of both maximal and sustainable oxidative metabolism in highly trained female middle‐distance runners.

## AUTHOR CONTRIBUTIONS

**Mira I. Schoeberlein:** Conceptualization; data curation; formal analysis; investigation; methodology; project administration; visualization. **Louis D. Hunter:** Data curation; formal analysis; investigation; methodology; project administration. **Emma W. Richardson:** Data curation; formal analysis; investigation; methodology; visualization. **Brian A. Irving:** Formal analysis; investigation; validation. **Brad W. Wilkins:** Conceptualization; data curation; formal analysis; funding acquisition; investigation; methodology; project administration; validation; visualization.

## FUNDING INFORMATION

Funding for this study was provided in part by Nike Inc.

## CONFLICT OF INTEREST STATEMENT

E.W.R. holds stock in Nike Inc. There are no other potential conflicts of interest. Nike Inc. had no role in the study design, data collection, data analysis, interpretation of the results, manuscript preparation, or conclusions of this study. The results of the study are presented clearly, honestly, and without fabrication, falsification, or inappropriate data manipulation.

## ETHICS STATEMENT

The Institutional Review Board at the University of Oregon approved this study. Written informed consent was obtained from all participants, and the study conformed to the principles outlined in the Declaration of Helsinki.
